# Circulating CD24/Siglec-10 biomarkers predict post-resuscitation outcomes in a cardiac arrest cohort

**DOI:** 10.1038/s41598-025-21775-z

**Published:** 2025-10-29

**Authors:** Ying Liu, Yushu Chen, Ling Wang, Dongping Yu, Peiyan Chen, Shaolin Chen, Ping Gong

**Affiliations:** 1 Department of Emergency Medicine, First Affiliated Hospital of Dalian Medical University, Dalian, Liaoning China; 2 Department of Emergency Medicine, Shenzhen People’s Hospital (The Second Clinical Medical College, Jinan University; The First Affiliated Hospital, Southern University of Science and Technology), Shenzhen, Guangdong China; 3 Department of Critical Care Medicine, Affiliated Hospital of Zunyi Medical University, Zunyi, Guizhou China; 4 Department of Emergency Medicine, Second Affiliated Hospital of Dalian Medical University, Dalian, Liaoning China

**Keywords:** Cardiac arrest, Return of spontaneous circulation, sCD24, Siglec-10, Prognosis, Prognostic markers, Hypoxic-ischaemic encephalopathy

## Abstract

CD24 can bind to its receptor, Siglec-10, and suppress immune responses induced by tissue damage. We aimed to study serum biomarkers in CD24/Siglec-10 axis and explore their associations with the survival and neurological prognosis of patients after return of spontaneous circulation (ROSC) following cardiac arrest. A prospective cohort study was performed. Eligible patients with ROSC were enrolled. Clinical information and serum levels of sCD24, sSiglec-10, sialic acid, tumor necrosis factor (TNF-α), interleukin-6 (IL-6), high mobility group protein 1 (HMGB1), neuron specific enolase (NSE), and neuraminidase activity were collected on days 1, 3 and 7 after ROSC. The 28-day survival and neurological outcome were documented. The study included 104 ROSC patients, with 30 and 74 in survivor and non-survivor groups, respectively. The levels of all biomarkers, except for sSiglec-10, sialic acid, and neuraminidases on day 1, were significantly higher in non-survivor than survivor group. Serum sCD24 level was positively correlated with sialic acid, HMGB1, TNF-α, IL-6, NSE, neuraminidases activity, and APACHE II score. Multivariate logistic regression analysis showed that serum sCD24 level was independently associated with 28-day poor neurological prognosis and mortality after ROSC. Multiple serum biomarkers within the CD24/Siglec-10 axis were significantly elevated following ROSC. Elevated serum sCD24 level emerged as a predictor of both 28-day poor neurological prognosis and all-cause mortality in patients after cardiac arrest. Further large-scale studies are warranted to validate these findings.

**Supplementary Information:**

The online version contains supplementary material available at 10.1038/s41598-025-21775-z.

## Introduction

Even with extensive research on the cardiopulmonary resuscitation (CPR), a significant number of patients still suffered from poor prognosis following cardiac arrest (CA)^1,2^. The average global survival rate to hospital discharge was 8.8% among these affected patients^1^. In China, this rate was only approximately 1.15%^3^. According to a Baseline Investigation of Out-of-Hospital CA survey conducted in 2023, as low as 0.83% patients could have satisfactory neurological outcomes after CA^3^. A complex pathophysiological process known as post-cardiac arrest syndrome (PCAS) might contribute to the low survival rate in these patients^4^. This syndrome encompasses post-CA brain damage, myocardial dysfunction, systemic ischemia/reperfusion response, and persistent precipitating pathology^4^. It was reported that, following return of spontaneous circulation (ROSC), a systemic ischemia/reperfusion injury could trigger the systemic inflammatory activation resembling the process of sepsis, which was characterized by elevated levels of inflammatory cytokines, such as interleukin (IL)-6 and tumor necrosis factor (TNF)-α, without the presence of endotoxin in plasma^5^. TNF-α and IL-6 are early-response pro-inflammatory cytokines with significantly elevated expression levels in the initial stage after brain injury^6–8^. Their high levels have been associated with adverse and severe neurological outcomes^9^. In addition, our previous study also found the increased plasma levels of IL-6 and TNF-α after ROSC^10^, indicating that pro-inflammatory response could be activated after ROSC.

Cluster of differentiation 24 (CD24) is a sialylated glycoprotein located at the membranes of various tumor and hematopoietic cells. It can bind to sialic acid-binding immunoglobulin-like lectin (Siglec)-10 to function as an emerging immune checkpoint^11–13^. Siglecs are found on the majority of white blood cells to participate in multiple immune cell signaling pathways. Siglecs can distinguish non-self molecules by recognizing sialic acid-containing glycans in the glycoproteins and glycolipids^14^. The CD24-Siglec interaction has been recognized as an innate immune checkpoint that can modulate the host response after tissue injuries^11–13^ and cancer immunotherapy^15^. In addition, the CD24-Siglec interaction has been demonstrated to selectively suppress certain host tissue damage-caused immune responses, including inflammatory reactions to damage-associated molecular patterns (DAMP) but not to microbial pathogen-associated molecular patterns (PAMP), and macrophage-mediated phagocytosis (here CD24/Siglec-10 as “do not eat me” signal)^13,16,17^. During the DAMP-induced inflammation, CD24 forms a complex with Siglec-10 and DAMP such as high-mobility group box-1 (HMGB1), thereby suppressing the activation of pro-inflammatory HMGB1/Toll-like receptor (TLR)4/NF-κB axis and its downstream pro-inflammatory cytokines, such as IL-6 and TNF-α^11,13^.

A arginine residue in the V-set domain of Siglec-10 is involved in sialic acid recognition that is responsible for the binding of CD24 with Siglec-10^11,13^. Sialidases, also known as neuraminidases, can hydrolyze the sialic acid fragments within the extracellular domain of CD24, leading to de-sialylation and thereby preventing the interaction between CD24 and Siglec G (Siglec-10 in humans) in an animal study^12,18^. In mammals, four sialidase isoforms have been identified, each with distinct substrate specificity and cellular distribution designated as neuraminidase-1, 2, 3 and 4. Up-regulation of neuraminidase-1 following myocardial ischemia-reperfusion could lead to inflammation exacerbation by activating monocytes/macrophage invasion, ultimately causing myocardial damage and cardiac dysfunction^19^. Conversely, sialidase inhibitors could inhibit the neuraminidases-mediated CD24 desialylation and protect mice from sepsis^12^.

Consequently, both DAMPs and CD24/Siglec-10 axis could play an important role in regulating the immune response after tissue injury. CD24 binds to Siglec-10 and regulates excessive DAMP-mediated inflammation by suppressing the inflammatory response. This effect can be reversed by the CD24 desialylation mediated by sialidase. Studies have indicated that soluble CD24 (sCD24) could be significantly elevated in patients with aseptic inflammation resulting from the active rheumatoid arthritis^20^. CD24Fc, a form of soluble CD24, can attenuate the extensive inflammatory response triggered by damage-associated molecular patterns. This is achieved by binding to HMGB1 and heat shock proteins and regulating the downstream Siglec10-Src homology 2 domain-containing phosphatase 1 pathway^21^. Moreover, in patients with subarachnoid hemorrhage, soluble Siglec-10 (sSiglec-10) in the cerebrospinal fluid could increase rapidly^22^. Thus, we hypothesized that sCD24 and sSiglec-10 might be implicated in regulating the body’s inflammatory response process after ROSC. Unlike other neurological biomarkers such as neuron-specific enolase (NSE)^23^, the role of CD24 in hypoxic-ischemic encephalopathy has not been elucidated.

Therefore, we performed a prospective cohort study and aimed to examine the dynamic changes of the circulating protein biomarkers related to the CD24/Siglec-10 axis, such as sCD24, sSiglec-10, sialic acid, and the relative activity of sialidase, and investigate their associations with neurological prognosis and all-cause mortality in patients after ROSC.

## Methods

### Study design

We conducted a prospective cohort study at the cardiac intensive care unit (ICU) and emergency ICU in the First Affiliated Hospital and the Second Affiliated Hospital of Dalian Medical University at Dalian, China, between January 2022 and December 2023. This study protocol was approved by the Medical Ethics Committee of the First Affiliated Hospital of Dalian Medical University (PJ-KS-KY-2022-256) and followed the principles of the Declaration of Helsinki (2013 edition)^24^. The written informed consent was obtained from the legal guardians of the patients.

### Participant selection criteria

The inclusion criteria were patients ≥ 18 years old and successfully resuscitated from CA. The exclusion criteria were patients with severe infections, autoimmune diseases, long-term use of immunosuppressive agents or hormones, malignant tumors, pregnancy or lactation, as well as with other severe systemic conditions, such as hematological diseases and liver cirrhosis. Enrolled patients were stratified into two groups (survivors and non-survivors) based on their 28-day survival outcome. Considering the baseline values of the patients before CA were unavailable, we recruited age- and sex-matched healthy volunteers to explore the changes of circulating protein biomarkers related to the CD24/Siglec-10 axis after CA.

### Data collections

All enrolled patients received treatments according to the 2020 International Consensus on CPR^25^, except the targeted temperature management. The research assistants collected the clinical data prospectively, including demographic information, medical history, length of ICU stay, causes of CA, initial cardiac rhythm, witnessed CA events, bystander CPR, CPR duration, treatments, laboratory findings, and survival or death.

Within 24 h after ROSC, for patients with in-hospital cardiac arrest (IHCA), the “Good Outcome after Attempted Resuscitation (GO-FAR) 2 score”, which is used to predict neurological outcomes following IHCA^26^, was collected. For patients with out-of-hospital cardiac arrest (OHCA), the “MIRACLE_2_ score”, employed for the early prediction of poor neurological outcomes^27^, was also obtained. The Acute Physiology and Chronic Health Evaluation (APACHE II) scores were calculated on days 1, 3, and 7 after ROSC. The neurological prognosis on day 28 after ROSC was assessed by a cerebral performance category (CPC) score. A CPC score of 1–2 or 3–5 indicated a favorable or unfavorable neurological outcome, respectively.

### Serum biomarker measurements

The peripheral venous blood specimens were collected within 2 h (day 1), at 72 h (day 3), and 168 h (day 7) after ROSC or upon enrollment for healthy volunteers. After centrifugation at 1,000 *g* for 15 min at 4 °C, the isolated sera were subjected to the enzyme-linked immunosorbent assay to detect serum sCD24 (Fine Test, Wuhan, China), sialic acid (Fine Test, Wuhan, China), sSiglec-10 (Fine Test, Wuhan, China), IL-6 (Elabscience, Wuhan, China), total NSE (CUSABIO, Wuhan, China), HMGB1 (Elabscience, Wuhan, China), and TNF-α (Elabscience, Wuhan, China) according to the manufacture instructions. The relative activity of neuraminidases (Beyotime, Shanghai, China) was measured using fluorometry (BioTek Synergy H1, USA). According to the manufacture instructions, the detection parameters were set at the excitation/emission wavelengths of 322/450 nm. The relative neuraminidase activity among samples was calculated based on the detected fluorescence intensity.

### Statistical analyses

The data analyses and graphing were performed in SPSS v26 (IBM, Armonk, NY) and GraphPad Prism 10 (GraphPad Software Inc, La Jolla, Calif). The sample size was calculated based on preliminary data from our pilot study that included 18 patients (9 survivors and 9 non survivors). We selected an effect size of d = 1.5 (since Cohen’s d values ranged from 1.78 to 4.03) for the sCD24 difference between the survival and non-survival groups. With α = 0.017 (Bonferroni-adjusted for 3 comparisons) and a power = 90%, the minimum sample size for the survival group and the non-survival group at the three time points was 13 (a total of 78 samples across 6 groups). Considering clinical variability and potential attrition, we targeted to enroll a total of 116 patients with ROSC into the study.

The continuous data were presented as means ± standard deviations or median with interquartile range (IQR), depending on the normality test results. The categorical data were presented as numbers (percentage). Depending on the normality test results, the continuous variables at different time points among the survivors, non-survivors, and healthy volunteers were compared by the repeated-measures analysis of variance (ANOVA) or Kruskal-Wallis one-way ANOVA, which was followed by Bonferroni corrections for multiple comparisons. Two-group comparisons were completed by the Student *t* test or Mann-Whitney *U* test. The categorical variables were compared by the Chi-squared (or Fisher’s exact) test. The associations between sCD24 levels and other variables were evaluated by the Spearman’s correlation test. Receiver operating characteristic (ROC) curves were generated to investigate the predictive performance of the serum levels of sCD24, sSiglec-10, sialic acid, and the relative activity of neuraminidases for 28-day all-cause mortality or poor neurological prognosis. The areas under the curves (AUCs) were presented and compared using DeLong’s test. According to the AUCs, the most predictive indicator (sCD24) in the CD24/Siglec-10 axis was selected to calculate the optimal thresholds, specificity, sensitivity, negative predictive value (NPV), positive predictive value (PPV), negative likelihood ratio (LR-), positive likelihood ratio (LR+), and Youden index. Then, to study the relationship between serum sCD24 and 28-day neurological prognosis or all-cause mortality after ROSC, multivariate logistic regression analyses were conducted. The odds ratios (OR) with 95% confidence intervals (CI) were reported. A *P* < 0.05 was considered statistically significant.

## Results

### Participant enrollment and baseline characteristics

**Fig. 1:**
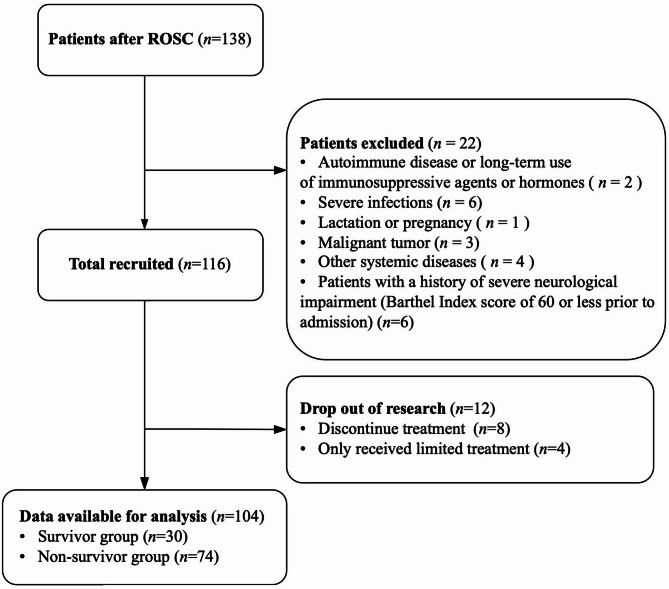
Participant enrollment flowchart. ROSC, restoration of spontaneous circulation.

A total of 104 patients with CA were included in the final analysis, including 30 and 74 in the survivor and non-survivor groups, respectively (Fig. 1). Due to the unavailability of baseline values for circulating protein biomarkers related to the CD24/Siglec-10 axis prior to CA, 32 healthy volunteers matched for sex and age were enrolled as a control group. No statistically significant differences in sex or age were observed among the control group, survival group, and non-survival group (all *P* > 0.05). As shown in Table 1, witness CA, bystander CA, and shockable rhythm events were less common and length of ICU stay was shorter in the non-survivor group compared with the survivor group. Compared with the non-survivors, the survivors had a significantly shorter CPR duration and a lower APACHE II score. 60% (18/30) of the survival patients had a favorable neurological prognosis with CPC scores of 1–2 at day 28. In addition, the baseline lactate and creatinine were significantly elevated in the non-survivor group compared to those in the survivor group. Hemodynamic support therapy was more frequently administered to patients in the non-survivor group than the survivor group. Other characteristics were comparable between survivors and non-survivors.

**Table 1:** Baseline characteristics of the study participants.

Characteristics	Healthy volunteers(*n* = 32)	Survivors(*n* = 30)	Non-survivors(*n* = 74)	^1^*P* values
Age, years	67.0 (52.5, 74.8)	69.5 (65.3, 78.5)	68.0 (51.0, 79.0)	0.706
Male, *n* (%)	18 (56.3%)	16 (53.3%)	47 (63.5%)	0.380
Previous medical history, *n* (%)				
Diabetes	–	6 (20.0%)	20 (27.0%)	0.618
Hypertension	–	16 (53.3%)	43 (58.1%)	0.669
Coronary heart disease	–	14 (46.7%)	29 (39.2%)	0.515
Chronic pulmonary disease	–	3 (10.0%)	8 (10.8%)	1.000
Chronic kidney disease	–	3 (10.0%)	10 (13.5%)	0.752
Post-operation	–	5 (16.7%)	11 (14.9%)	0.773
Causes of CA, *n* (%)				
Cardiac	–	11 (36.7%)	33 (44.6%)	0.516
Respiratory	–	10 (33.3%)	20 (27.0%)	0.663
Cerebral	–	4 (13.3%)	9 (12.2%)	1.000
Others	–	5 (16.7%)	12 (16.2%)	1.000
Location of CA, *n* (%)				
Out-of-hospital	–	8 (26.7%)	25 (33.8%)	0.642
In-hospital	–	22 (73.3%)	49 (66.2%)	0.632
Witnessed CA, *n* (%)	–	27 (90.0%)	49 (66.2%) ^#^	0.015
Bystander CPR, *n* (%)	–	28 (93.3%)	50 (67.6%) ^#^	0.006
Initial cardiac rhythm, *n* (%)				
Shockable rhythm	–	19 (63.3%)	19 (25.7%) ^#^	0.001
Non-shockable rhythm	–	11 (36.7%)	55 (74.3%) ^#^	0.001
CPR time, minutes	–	8.0 (5.0, 10.0)	15 (10.0, 20.0) ^#^	< 0.001
Length of ICU stay, days	–	10.5 (7.5, 17.0)	3.0 (1.0, 5.0) ^#^	< 0.001
Treatments, *n* (%)				
Ventilator use	–	30 (100%)	74 (100%)	–
Sedation	–	30 (100%)	74 (100%)	–
Hemodynamic support	–	14 (46.6%)	74 (100%) ^#^	< 0.001
Seizure treatment	–	4 (13.3%)	11 (14.7%)	1.000
Laboratory results				
Procalcitonin, ng/mL		1.26 (0.16, 4.20)	0.73 (0.11, 4.89)	0.825
Lactate, mmol/L		2.65 (1.76, 3.62)	6.20 (3.40, 12.80) ^#^	< 0.001
Creatinine, mmol/L		78.5 (60.3, 92.8)	109.0 (86.5, 171.5) ^#^	< 0.001
hs-troponin I, mg/L		0.621 (0.049, 2.205)	0.612 (0.156, 1.618)	0.446
BNP, pg/mL		140.7 (87.7, 296.9)	294.8 (88.1, 774.0)	0.082
APACHE II score	–	19.5 (16.0, 22.3)	32 (27.0, 32.8) ^#^	< 0.001
28-day CPC (1–2), *n* (%)	–	18 (60.0%)	–	

All data are presented as median (interquartile range), unless otherwise specified. ^*1*^*P* values for the comparison of non-survivors compared with survivors. ^#^*P* < 0.05 compared with survivors. *APACHE II* acute physiology and chronic health evaluation II, *BNP* brain natriuretic peptide, *CPC* cerebral performance category, *CA* cardiac arrest, *CPR* cardiopulmonary resuscitation, *hs-troponin I* high-sensitivity troponin-I, *ICU* intensive care unit, *WBC* white blood cell.

### Comparison of serum levels of sCD24, sSiglec-10, sialic acid, IL-6, the relative activity of neuraminidases, HMGB1, and TNF-α

**Fig. 2:**
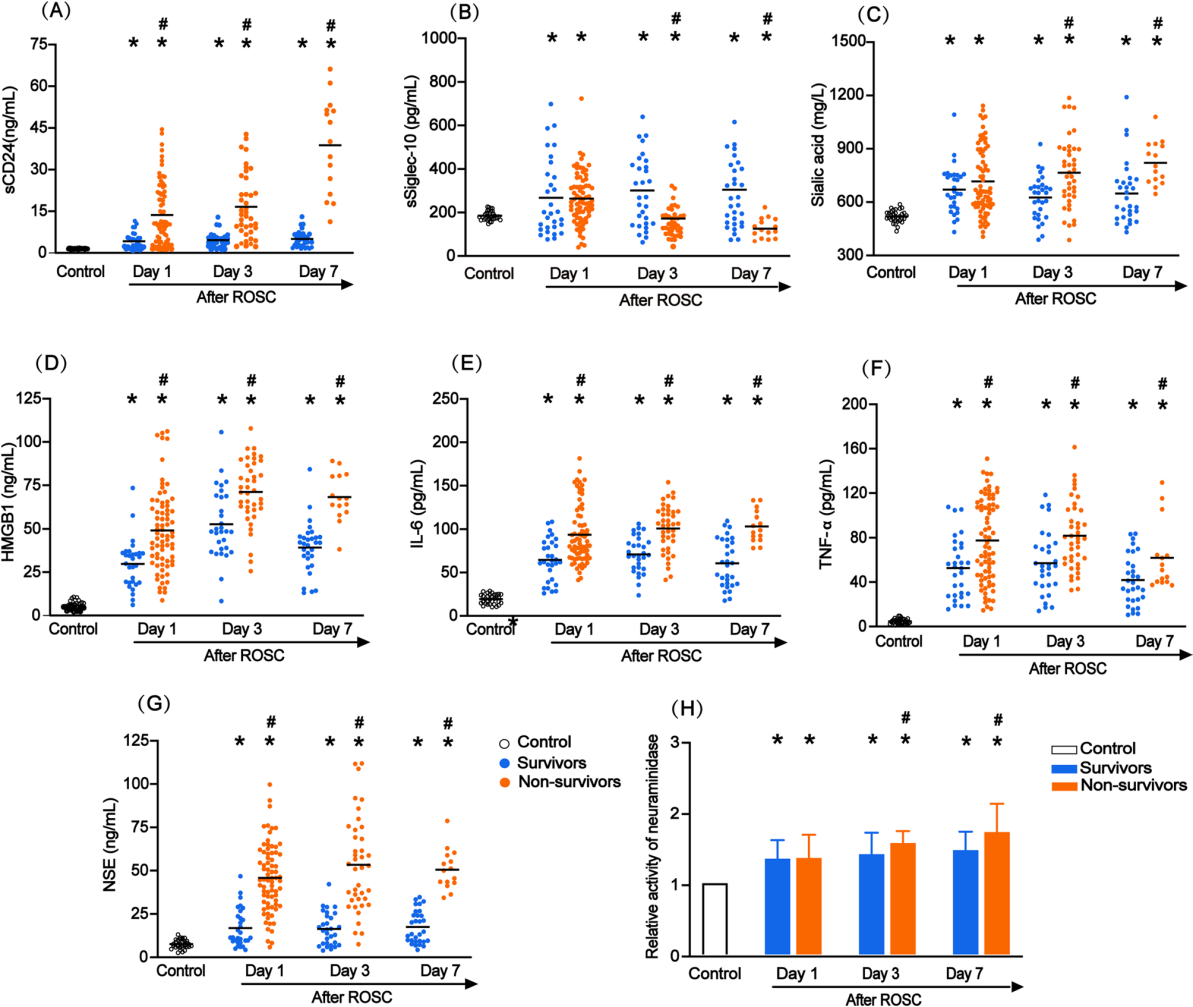
Comparisons of serum levels of sCD24 (**A**), sSiglec-10 (**B**), Sialic acid (**C**), HMGB1 (**D**), IL-6 (**E**), TNF-α (**F**), NSE (**G**), and the relative activity of neuraminidases (**H**) among healthy volunteers (*n* = 32), survivors (*n* = 30), and non-survivors (*n* = 74). ^*^P < 0.05 compared with healthy volunteers; ^#^*P* < 0.05 compared with survivors. HMGB1, high-mobility group box-1; IL-6, interleukin-6; NSE, neuron specific enolase; ROSC, restoration of spontaneous circulation; sCD24, soluble cluster of differentiation 24; sSiglec-10, soluble sialic acid-binding immunoglobulin-like lectin-10.

On day 1, 3, and 7 after ROSC, the patients exhibited significantly higher levels of serum sCD24, sSiglec-10, sialic acid, IL-6, HMGB1, TNF-α, and NSE as well as the relative activity of neuraminidases than the healthy volunteers (Fig. 2). In addition, serum sCD24, HMGB1, IL-6, TNF-α, and NSE levels were significantly higher in the non-survivors than survivors on day 1, 3, and 7 after ROSC (Fig. 2). However, the serum sialic acid levels and the relative activities of neuraminidases were significantly higher in the non-survivors than the survivors only on day 3 and 7 after ROSC (Fig. 2). Significant decreases in serum sSiglec-10 levels were also observed in the non-survivors than the survivors on day 3 and 7 after ROSC (Fig. 2).

### Correlations of serum sCD24 with sSiglec-10, sialic acid, relative activity of neuraminidases, HMGB1, IL-6, TNF-α, NSE and APACHE II score

On day 1, 3, and 7 after ROSC, the serum sCD24 levels were positively correlated with serum sialic acid, the relative activity of neuraminidase, HMGB1, IL-6, TNF-α, NSE, and the APACHE II score (all *P* < 0.05, Supplementary Table S1). It should be noted that, on day 1, the correlations between sCD24 and sialic acid, neuraminidase, and the APACHE II score were relatively weak (*r* < 0.3). On day 3 and 7, sCD24 exhibited strong correlations with these biomarkers (*r* > 0.5). sSiglec-10 was only weakly positively correlated with sCD24 on day 1 (*P* < 0.05, *r* = 0.305).

### Performance of sCD24 level in predicting 28-day all-cause mortality

**Fig. 3:**
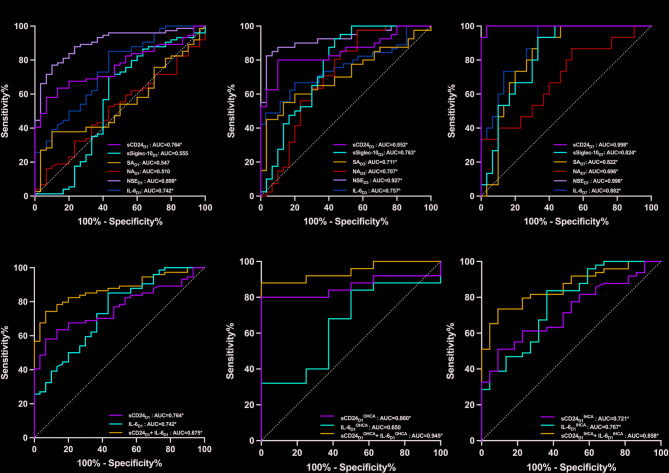
Receiver operating characteristic curves of variables for predicting 28-day all-cause mortality following ROSC. *P < 0.05; AUC, areas under the curves; IHCA, in-hospital cardiac arrest; IL-6_D1,D3,D7_, interleukin-6 on day 1, 3 and 7 after ROSC; NA_D1,D3,D7_, the relative activity of neuraminidase on days 1, 3 and 7 after ROSC; NSE_D1,D3,D7_, neuron specific enolase on days 1, 3 and 7 after ROSC; OHCA, out-of-hospital cardiac arrest; sCD24_D1, D3,D7_, soluble cluster of differentiation 24 on days 1, 3 and 7 after ROSC; SA_D1,D3,D7_, Sialic acid on days 1, 3 and 7 after ROSC; sSiglec-10_D1,D3,D7_, soluble Siglec-10 on days 1, 3 and 7 after ROSC.

Serum sCD24 levels on days 1, 3, and 7 after ROSC could predict 28-day all-cause mortality. Additionally, sSiglec-10 and sialic acid levels, as well as the relative activity of neuraminidase on days 3 and 7, also showed predictive value for 28-day all-cause mortality (all *P* < 0.05; Fig. 3A–C; Table 2). The serum sCD24 level on day 7 had the highest predictive performance (AUC = 0.998, 95% CI: 0.991–1.000), sensitivity, and specificity (Table 3). Table 3 summarizes the AUC, cut-off values, sensitivity, specificity, PPV, NPV, Youden index, and likelihood ratios for each variable.

**Table 2:** Areas under the curve of various parameters for predicting 28-day all‐cause mortality or poor neurological prognosis in patients after ROSC.

	28-day all-cause mortality			28-day poor neurological prognosis		
	AUC	95%CI	*P*	AUC	95% CI	*P*
sCD24_D1_	0.764	0.675–0.853	0.000	0.812^B^	0.726–0.897	0.000
sCD24_D3_	0.852	0.762–0.942	0.000	0.814^C^	0.717–0.912	0.000
sCD24_D7_	0.998	0.991-1.000	0.000	0.819^D^	0.695–0.943	0.000
sSiglec-10_D1_	0.555	0.414–0.695	0.385	0.549	0.381–0.717	0.517
sSiglec-10_D3_	0.763	0.641–0.883	0.000	0.841	0.739–0.943	0.000
sSiglec-10_D7_	0.824	0.706–0.943	0.000	0.722	0.573–0.871	0.012
Sialic acid_D1_	0.547	0.433–0.662	0.451	0.578	0.446–0.709	0.303
Sialic acid_D3_	0.711	0.590–0.831	0.003	0.672	0.542–0.802	0.031
Sialic acid_D7_	0.822	0.700-0.944	0.001	0.722	0.573–0.871	0.012
Neuraminidase_D1_	0.510	0.392–0.627	0.877	0.545	0.410–0.660	0.550
Neuraminidase_D3_	0.707	0.575–0.838	0.003	0.692	0.531–0.853	0.016
Neuraminidase_D7_	0.696	0.528–0.864	0.034	0.613	0.449–0.778	0.203
NSE_D1_	0.899	0.838–0.960	0.000	0.853	0.780–0.927	0.000
NSE_D3_	0.927	0.865–0.989	0.000	0.873	0.780–0.954	0.000
NSE_D7_	0.998	0.991-1.000	0.000	0.870	0.760–0.981	0.000
IL-6_D1_	0.742	0.638–0.846	0.000	0.711	0.599–0.824	0.005
IL-6_D3_	0.757	0.652–0.862	0.000	0.712	0.592–0.831	0.008
IL-6_D7_	0.882	0.787–0.978	0.000	0.650	0.489–0.812	0.091
sCD24_D1_+ IL-6_D1_	0.875^Aa^	0.810–0.940	0.000	–	–	–
sCD24_D1_ + NSE_D3_	–	–	–	0.938^Ee^	0.883–0.993	0.000

^A^*P*=0.004 (*Z* = 2.902) vs. sCD24_D1_; ^a^*P*=0.002 (*Z* = 3.165) vs. IL-6_D1_; ^B^*P*=0.904 (*Z*=-2.78) vs. NSE_D1_; ^C^*P*=0.284 (*Z*=-1.071) vs. NSE_D3_; ^D^*P*=0.242 (*Z*=-1.171) vs. NSE_D7_; ^E^*P*=0.025(*Z* = 2.236) vs. sCD24_D1_; ^e^*P*=0.039 (*Z* = 2.061) vs. NSE_D3_; *AUC* areas under the curve, *CI* confidence interval, *IL-6*_*D1*_, _*D3*_, _*D7*_ interleukin-6 on day 1, 3 and 7 after ROSC, *Neuraminidase*_*D1*_, _*D3*_, _*D7*_ the relative activity of neuraminidase on days 1, 3 and 7 after ROSC, *NSE*_*D1*_, _*D3*_, _*D7*_ neuron specific enolase on days 1, 3 and 7 after ROSC, *ROSC* return of spontaneous circulation, *sCD24*_*D1*_, _*D3*_, _*D7*_ soluble cluster of differentiation 24 on days 1, 3 and 7 after ROSC, *Sialic acid*_*D1*_, _*D3*_, _*D7*_ Sialic acid on days 1, 3 and 7 after ROSC, s*Siglec-10*_*D1*_, _*D3*_, _*D7*_ soluble Siglec-10 on days 1, 3 and 7 after ROSC.

**Table 3:** Performance of variables after ROSC in predicting 28-day all-cause mortality or 28-day poor neurological prognosis.

	28-day all-cause mortality									28-day poor neurological prognosis							
	Cut-off (ng/mL)	Specificity (%)	Sensitivity (%)	NPV (%)	PPV (%)		LR–	LR+	Youden (%)	Cut-off (ng/mL)	Specificity (%)	Sensitivity (%)	NPV (%)	PPV (%)	LR–	LR+	Youden (%)
sCD24_D1_	9.33	93.3	58.1	47.5	95.6		0.45	8.67	51.4	9.33	94.4	51.2	28.8	97.8	0.52	9.14	45.6
sCD24_D3_	6.64	90.0	80.0	64.6	95.2		0.22	8.00	70.0	6.64	88.9	63.6	33.8	96.5	0.41	5.73	52.4
sCD24_D7_	10.81	96.7	100	100	98.6		0.00	30.03	96.7	10.81	100	59.3	34.0	100	0.41	–	59.3
NSE_D3_	29.70	96.7	82.5	69.0	98.4		0.18	25.0	79.2	29.70	100	65.4	37.7	100	0.35	–	65.4
IL-6_D1_	65.19	56.7	85.1	60.7	82.9		0.26	1.97	41.8	88.98	94.4	43.0	25.7	97.3	0.60	7.68	37.46
sCD24_D1_ + NSE_D3_	–	–	–	–	–		–	–	–	–	88.3	92.3	100	97.4	0.09	7.89	80.55
sCD24_D1_ + IL-6_D1_	–	93.33	74.32	59.6	96.5		0.28	11.1	67.65	–	–	–	–	–	–	–	–

*IL-6*_*D1*_ interleukin-6 on day 1 after ROSC, *LR*^*–*^ negative likelihood ratio, *LR*^*+*^ positive likelihood ratio, *NPV* negative predictive value, *PPV* positive predictive value, *NSE*_*D3*_ neuron specific enolase on days 3 after ROSC, *sCD24*_*D1*_, _*D3*_, _*D7*_ soluble cluster of differentiation 24 on days 1, 3 and 7 after ROSC.

sCD24 is known to suppress DAMP- and TLR-mediated activation, potentially reducing the release of inflammatory factors such as IL-6 and TNF-α. As an early-response cytokine, IL-6 increases markedly following brain injury. Given their complementary roles, we tested whether combining sCD24 with IL-6 would improve prognostic accuracy for 28-day all-cause mortality after ROSC. The results showed that combination of day 1 sCD24 and IL-6 achieved a higher AUC (0.875, 95% CI: 0.810–0.940) compared to sCD24 (0.764, 95% CI: 0.675–0.853, *P* = 0.004) or IL-6 alone (0.742, 95% CI: 0.638–0.846, *P* = 0.002) (Fig. 3D; Table 2), indicating enhanced predictive value.

We further stratified patients into OHCA and IHCA subgroups. No significant differences were observed in sex, age, or initial rhythm between the subgroups (Supplementary Table S2). Day 1 sCD24 predicted 28-day all-cause mortality in both subgroups (all *P* < 0.05; Fig. 3E,F). However, day 1 IL-6 did not show significant predictive ability in the OHCA subgroup (Fig. 3E), which might be attributable to the relatively small sample size of OHCA patients in our study. Notably, the combination of day 1 sCD24 and IL-6 improved predictive performance for 28-day all-cause mortality in both subgroups compared to either biomarker alone (Fig. 3E,F, Supplementary Table S3). Supplementary Table S3 provides detailed prognostic performance values for sCD24 on days 1, 3, and 7.

### Performance of sCD24 level in predicting 28-day poor neurological prognosis

**Fig. 4:**
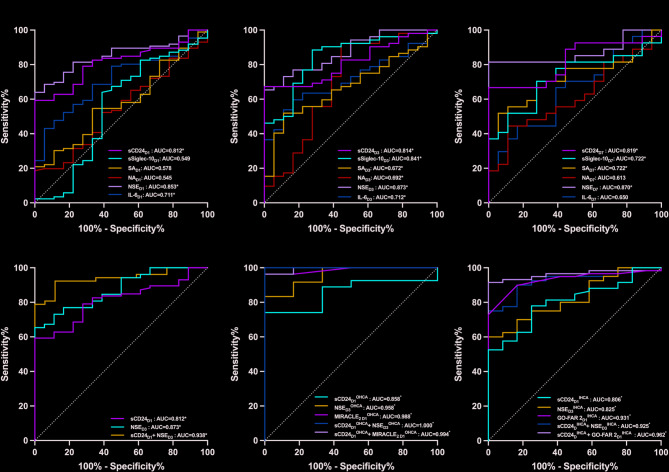
Receiver operating characteristic curves of variables for predicting 28-day poor neurological prognosis following ROSC. ^*^P < 0.05; AUC, areas under the curves; GO-FAR 2, the Good Outcome Following Attempted Resuscitation (GO-FAR) 2 score; IHCA, in-hospital cardiac arrest; IL-6_D1,D3,D7_, interleukin-6 on day 1, 3 and 7 after ROSC; MIRACLE_2_, a risk score for early prediction of neurological outcome after out-of-hospital cardiac arrest; NA_D1,D3,D7_, the relative activity of neuraminidase on days 1, 3 and 7 after ROSC; NSE_D1,D3,D7_, neuron specific enolase on days 1, 3 and 7 after ROSC; OHCA, out-of-hospital cardiac arrest; sCD24_D1, D3,D7_, soluble cluster of differentiation 24 on days 1, 3 and 7 after ROSC; SA_D1,D3,D7_, Sialic acid on days 1, 3 and 7 after ROSC; sSiglec-10_D1,D3,D7_, soluble Siglec-10 on days 1, 3 and 7 after ROSC.

Serum sCD24 measured on days 1, 3, and 7 after ROSC demonstrated predictive value for 28-day poor neurological prognosis. Similarly, sSiglec-10 and sialic acid levels on days 3 and 7, as well as neuraminidase activity on day 3, were also predictive for 28-day poor neurological prognosis (all *P* < 0.05; Fig. 4A–C; Table 2). The day 7 sCD24 level had the highest predictive performance (AUC = 0.819, 95% CI: 0.695–0.943), sensitivity, and specificity (Table 3). Detailed prognostic performance values of sCD24 are provided in Table 3.

NSE is an established biomarker for neuronal injury and poor neurological prognosis after CA, with peak levels at 48–72 h post-ROSC offering the most significant prognostic value^28^. In this study, sCD24 showed predictive performance comparable to NSE at each time point (days 1, 3, and 7) for 28-day poor neurological outcome (Table 2). Given the potential role of sCD24 in modulating systemic inflammation and mitigating neuroinflammation following ROSC, we evaluated the combined use of day 1 sCD24 and day 3 NSE to predict 28-day neurological prognosis, which yielded an AUC of 0.938 (95% CI: 0.883–0.993), significantly surpassing day 1 sCD24 (AUC = 0.812, 95% CI: 0.726–0.897, *P* = 0.025) and day 3 NSE alone (AUC = 0.873, 95% CI: 0.780–0.954, *P* = 0.039) (Fig. 4D; Table 2), indicating a substantial improvement in predictive performance.

Subgroup analyses of OHCA and IHCA patients revealed that both day 1 sCD24 (OHCA: AUC = 0.858, 95% CI: 0.729–0.987; IHCA: AUC = 0.806, 95% CI: 0.695–0.917) and day 3 NSE (OHCA: AUC = 0.958, 95% CI: 0.873–1.000; IHCA: AUC = 0.825, 95% CI: 0.711–0.939) independently predicted 28-day poor neurological prognosis. The combination of day 1 sCD24 and day 3 NSE further enhanced predictive performance, achieving an AUC of 1.000 (95% CI: 1.000–1.000) in the OHCA subgroup and 0.925 (95% CI: 0.853–0.997) in the IHCA subgroup (all *P* < 0.05; Fig. 4E,F).

The MIRACLE₂ and GO-FAR 2 scores are widely used predictive models for neurological prognosis in OHCA and IHCA patients, respectively^26,27^. We collected these scores (Supplementary Table S2) and performed further analyses. In OHCA patients, combining sCD24 with the MIRACLE₂ score resulted in superior predictive performance compared to either measure alone (AUC = 0.994, 95% CI: 0.974–1.000; Fig. 4E). Similarly, for IHCA patients, the combination of sCD24 and the GO-FAR 2 score yielded the highest AUC (0.962, 95% CI: 0.919–1.000; Fig. 4F) than each individual measurement. These findings suggested that integrating sCD24 with established biomarkers or clinical scores significantly enhanced the prediction of neurological outcomes in cardiac arrest patients. Detailed prognostic performance is listed in Supplementary Table S4.

### Independent predictors of 28-day all-cause mortality and poor neurological prognosis: multivariate logistic regression analysis

Variables with a *P* value less than 0.1 in univariate analysis were applied for multivariate analysis. We included sCD24 on day 1, shockable rhythm, CPR time, bystander CPR, and age > 60 years as the independent variables and 28-day mortality or neurological prognosis as the dependent variables in multivariate binary logistic regression analyses due to the limited sample size (Table 4). The results showed that serum sCD24 on day 1 was significantly associated with 28-day all-cause mortality (OR 1.212, 95% CI: 1.046–1.404, *P* = 0.010) and 28-day poor neurological prognosis (OR 1.518, 95% CI: 1.097–2.101, *P* = 0.012) after adjusting for other variables (Table 4).

**Table 4:** Univariate and multivariable logistic regression analysis on variables affecting 28-day all‐cause mortality and 28‐day poor neurological prognosis in patients after ROSC.

	28-day all‐cause mortality							28-day poor neurological prognosis					
	Univariate analysis			Multivariate analysis				Univariate analysis			Multivariate analysis		
	OR	95%CI	*P*	OR		95%CI	*P*	OR	95%CI	*P*	OR	95%CI	*P*
sCD24_D1_ (ng/mL)	1.211	1.046–1.402	0.011	1.212		1.046–1.404	0.010	1.601	1.104–2.323	0.013	1.518	1.097–2.101	0.012
Shockable rhythm	0.054	0.011–0.272	0.000	0.052		0.011–0.254	0.000	0.184	0.039–0.866	0.032	0.172	0.041–0.726	0.017
CPR time (minutes)	1.381	1.159–1.645	0.000	1.386		1.164–1.649	0.000	1.382	1.118–1.708	0.003	1.342	1.109–1.624	0.003
Age > 60 (years)	5.482	1.148–26.187	0.033	5.539		1.156–26.539	0.032	2.951	0.557–15.245	0.205			
Bystander CPR	0.766	0.098–5.967	0.799					0.837	0.056–10.349	0.837			

*CI* confidence interval, *OR* odd ratio, *ROSC* return of spontaneous circulation, *sCD24*_*D1*_ soluble cluster of differentiation 24 on day 1 after ROSC.

## Discussion

The pathophysiology of PCAS is highly complex, and the accurate prediction of prognosis in patients after CA remains a major clinical challenge. This study was the first research to investigate the dynamic changes and prognostic significance of sCD24 and related pathway components following CA. We observed marked and sustained increases in serum levels of sCD24 and sialic acid, as well as elevated neuraminidase activity, in non-survivors compared to survivors during the first week after ROSC. Elevated serum sCD24 levels were positively correlated with sialic acid, neuraminidase activity, proinflammatory mediators, and the neuronal injury marker NSE. Importantly, elevated sCD24 on day 1 after ROSC served as an independent predictor of 28-day all-cause mortality and poor neurological prognosis.

In the present study, we demonstrated that serum sCD24 remains persistently elevated during the first week after ROSC and serves as a reliable predictor of 28-day all-cause mortality. The exact mechanism by which CD24 is shed from the cell membrane has not yet been fully elucidated. CD24 is a highly glycosylated and dynamically expressed molecule present on diverse cells, including the immune cells, myeloid cells (mainly granulocytes), adipocytes, keratinocytes, microglia, astrocytes, and neurons^29^. After ROSC, the increased shed of CD24 from the cell membrane might be due to the potential enzymatic proteolysis, which can “cut-off” CD24 from the cell membrane, resulting in the release of CD24 into the extracellular space and subsequent activations of pro-inflammatory signaling pathways^30,31^. Another possibility is the increased expression of CD24 after exposure to ischemia or inflammatory insults after ROSC. The RNA and protein levels of CD24 were found to be markedly elevated in neurons, microglia, and astrocytes of the cerebral cortex after brain trauma in humans and mice in a time-dependent manner^32^. Recombinant HMGB1 has also been demonstrated to elicit increased mRNA and protein CD24 expressions on cultured astrocytes, which occurred at 24 h after stimulation and persisted for more than 48 h, accompanied by the activation of NF-κB signaling pathway^33^. In addition, recent studies showed the release of CD24 to extracellular space was likely to be achieved by exosomes^34,35^. Further analysis indicated that sCD24 was positively correlated with the levels of serum sialic acid, IL-6, HMGB1, TNF-α, and NSE, as well as the relative activity of neuraminidase. Additionally, it was positively correlated with the APACHE II score. Nevertheless, it should be noted that, on the first day, the correlations between sCD24 and sialic acid, neuraminidase, and the APACHE II score were relatively weak. This might be due to the fact that sCD24 cannot fully reflect the entire activity of CD24, and the early release or shedding of sCD24 might be relatively limited.

Following initial ischemic injury, the damaged cells could release DAMP molecules, such as HMGB1, which triggered a cascade of sterile inflammatory responses and stimulated excessive production of pro-inflammatory cytokines, including TNF-α and IL-6^36^. The CD24/Siglec-10 pathway has been documented to selectively repress the DAMP-associated inflammation by forming a trimolecular complex of CD24, Siglec-10, and HMGB1 to suppress the activation of HMGB1/NF-κB pro-inflammatory signaling pathway^11^. However, a positive correlation between serum sCD24 level and proinflammatory mediators (HMGB1, TNF-α, and IL-6) was observed here, which seemed to be contradictory to the above-mentioned association that CD24/Siglec-10 axis could inhibit the post-resuscitation excessive sterile inflammatory response. One plausible explanation is that elevated sCD24 under systemic hyperinflammation might represent a compensatory anti-inflammatory response, acting as an endogenous “braking” mechanism to counteract the ongoing cytokine storm^5,37^. Furthermore, the CD24 promoter region may contains potential NF-κB binding sites^38^, suggesting that NF-κB-mediated upregulation might contribute to increased CD24 expression and subsequent elevation of circulating sCD24. Thus, the correlation between sCD24 and pro-inflammatory cytokines likely indicated that sCD24 serves not as a direct driver of inflammation, but rather as a marker of a compensatory anti-inflammatory response, reflecting the severity of the initial inflammatory insult. Persistently high sCD24 levels may signal the development of a severe immunosuppressive state^39,40^, which has been linked to increased mortality in sepsis patients^41^. This assumption was further supported by our results that non-survivors exhibited significantly higher sCD24 levels on day 7 after ROSC compared to survivors. Furthermore, our findings suggested that combining sCD24 and IL-6 measurements on day 1 after ROSC could significantly enhance prognostic accuracy for mortality. IL-6, a key pro-inflammatory cytokine, rises markedly early after CA and has been associated with increased mortality^5,42^. Previous studies reported that treatment with tocilizumab — an IL-6 receptor antagonist — could attenuate the post-CA systemic inflammatory response and might help reduce organ damage^43^. The combination of IL-6 and sCD24 provides a more holistic view of the patient’s immune state, allowing for earlier identification of high-risk individuals.

Our results also demonstrate that post-ROSC sCD24 levels were predictive of 28-day poor neurological prognosis. Notably, the predictive performance of sCD24 was comparable to that of NSE—a widely accepted “gold standard” biomarker for neuronal injury—at each corresponding time point, indicating its potential value in prognostic assessment after CA. The mechanisms underlying post-CA brain injury are complex and extend beyond primary ischemic and hypoxic damage^44^. Secondary neuroinflammatory responses play a critical role in exacerbating neuronal injury. Following cerebral ischemia-reperfusion, activated microglia and astrocytes initiate central nervous system inflammation, disrupting neural structure and function, promoting neuronal death, and ultimately impairing neurological recovery^45–47^. The early elevation of serum sCD24 levels following resuscitation suggests an important role of the CD24/Siglec-10 signaling pathway in the negative regulation of both systemic and neuroinflammation. sCD24 may thus act as an early and sensitive marker reflecting the intensity of neuroinflammatory processes. Combining day 1 sCD24 with day 3 NSE provides a complementary assessment of neuroinflammation and structural neuronal injury, significantly improving the prediction of poor neurological prognosis.

Interestingly, serum sSiglec-10 levels remained consistently elevated in survivors on days 3 and 7 after ROSC, whereas they declined markedly in non-survivors during the same time period. This result conformed that CD24/Siglec-10 axis exerts the negative regulatory role in DAMP-induced inflammatory response, suggesting that the delayed decrease in serum sSiglec-10 levels was associated with the poor prognosis despite the presence of increased sCD24, which is similar to a previous study that a sustained elevation in Siglec-10 levels was related to a more favorable outcome in patients with intracranial hemorrhage from aneurysmal rupture^22^. Specifically, the decreased expression of sSiglec-10 in non-survivors after ROSC may lead to its failure to suppress the excessive inflammatory response induced by DAMP, resulting in the exacerbation of systemic inflammation and worse prognosis, but the reason for the decrease in sSiglec-10 levels remains unclear. However, various pathogens have been shown to disrupt CD24-Siglec-G/10 interactions mediated by down-regulation of Siglec G/10^48^. Given that secondary pathogen infections are often more severe following CPR in non-survivors after ROSC, it is possible that down-regulation of Siglec-10 may be associated with secondary infections following CPR and warrants further investigation.

Here, we identified a significant increase in serum sialic acid levels and the relative activity of neuraminidases after ROSC, suggesting a potential increase in tissue sialic acid and neuraminidases after ROSC. Neuraminidases (namely sialidases) are widely present in microorganisms, animals, and humans^49^. It is mainly located in the lysosomes, cytoplasmic vesicles, and cell membrane. The increase in the relative activity of neuraminidases after ROSC might be of microbial and/or host origin. The former is the release of neuraminidases from bacteria or virus in secondary infection, whereas the latter is due to the release of neuraminidases from injured cells after ROSC. The neuraminidases of host and microorganisms have similar functions^12^. Neuraminidases can hydrolyze the sialic acid fragment at the CD24 peptide extending out of the cell membrane. The desialylated CD24 loses its ability to interact with Siglec-10^12,18^. Therefore, the increase in serum neuraminidases is responsible for the removal of the CD24/Siglec-10 axis in repressing the DAMP-associated inflammation by hydrolyzing the sialic acid fragment of the extracellular domain of CD24 (CD24 desialylation). That is, the increase in serum neuraminidases could lead to, on the one hand, the increase in serum level of sialic acid, and on the one hand the exacerbation of inflammatory response that is associated with poor prognosis, as evidenced by our observation that the higher level of serum levels of sialic acid and the relative activity of neuraminidases in the non-survivors than the survivors. Therefore, it can be reasonably inferred that the increased sialidase activity leads to desialylation of CD24 on cell membranes, thereby attenuating the inhibitory effect of the CD24/Siglec-10 axis on the HMGB1/TLR4/NF-кB pathway, ultimately promoting neuroinflammation and exacerbating poor neurological prognosis.

It is worth noting that the efficacy of sialidase inhibitors, such as oseltamivir or zanamivir, in reducing sialic acid shedding and mitigating neutrophil overactivation has been demonstrated in COVID-19 patients, thereby attenuating the inflammatory response^50^. Soluble CD24 (CD24Fc), which is linked to the Fc domain of human IgG1, was developed to treat inflammation associated with viral infections, autoimmunity, and graft-versus-host diseases^51–53^. The modulation of CD24/Siglec-10 axis can hold promise in enhancing the neurological outcome of patients after ROSC.

This study has several limitations, including its small size and observational study design, with potential treatment variations in study participants. Therefore, the research results are exploratory. Furthermore, although there was a certain association between sCD24 and the aseptic inflammatory response following CA, given the intricate pathogenesis of post-CA syndrome, the predictive specificity of serum sCD24 for mortality and the prognosis of neurofunctional disorders in patients who have ROSC after CA could be limited. Moreover, the signaling mechanism of the soluble form may differ from that of the membrane-bound CD24/Siglec-10 interaction. As a result, sCD24 might not comprehensively reflect the entire functional state of the CD24/Siglec-10 axis, leading to limited predictive performance in the results. Therefore, additional studies with larger sample sizes are required to validate our findings herein.

## Conclusions

Circulating biomarkers of the CD24/Siglec-10 axis—including sCD24, sSiglec-10, sialic acid, and neuraminidase activity—were elevated following ROSC. Serum sCD24 demonstrated significant predictive value for 28-day all-cause mortality and poor neurological outcome after CA. The combination of day 1 sCD24 with either day 1 IL-6 or day 3 NSE improved early risk stratification and offered actionable clinical prognostic guidance. Further large-scale studies are warranted to validate these findings and to elucidate the functional role of the CD24/Siglec-10 axis in post-cardiac arrest patients.

## Supplementary Information

Below is the link to the electronic supplementary material.

Supplementary Material 1

Supplementary Material 2

Supplementary Material 3

Supplementary Material 4

## Data Availability

The datasets used in the present study are available from the corresponding author upon reasonable request.
