# Supplementary material for: Circulating CD24/Siglec-10 biomarkers predict post-resuscitation outcomes in a cardiac arrest cohort
Source: Sci Rep. 2025 Oct 29;15:37816. doi: 10.1038/s41598-025-21775-z (PMC12572198; doi:10.1038/s41598-025-21775-z)
Supplement: Supplementary file 1 — Supplementary Material 1 [file 41598_2025_21775_MOESM1_ESM.docx]

|  | **Serum sCD24** | |
| --- | --- | --- |
|  | *r* | *P* |
| **Day 1 after ROSC** |  |  |
| sSiglec-10 | 0.305 | 0.001 |
| HMGB1 | 0.737 | <0.001 |
| Sialic acid | 0.252 | 0.003 |
| Neuraminidase | 0.204 | 0.017 |
| IL-6 | 0.502 | <0.001 |
| TNF-α | 0.498 | <0.001 |
| NSE | 0.602 | <0.001 |
| APACHE II | 0.232 | 0.018 |
| **Day 3 after ROSC** |  |  |
| sSiglec-10 | -0.157 | 0.115 |
| HMGB1 | 0.687 | <0.001 |
| Sialic acid | 0.507 | <0.001 |
| Neuraminidase | 0.615 | <0.001 |
| IL-6 | 0.708 | <0.001 |
| TNF-α | 0.740 | <0.001 |
| NSE | 0.680 | <0.001 |
| APACHE II | 0.540 | <0.001 |
| **Day 7 after ROSC** |  |  |
| sSiglec-10 | -0.176 | 0.126 |
| HMGB1 | 0.840 | <0.001 |
| Sialic acid | 0.697 | <0.001 |
| Neuraminidase | 0.799 | <0.001 |
| IL-6 | 0.812 | <0.001 |
| TNF-α | 0.769 | <0.001 |
| NSE | 0.755 | <0.001 |
| APACHE II | 0.730 | <0.001 |

**Supplementary Table S1.** Correlations between serum sCD24 and different laboratory and clinical variables. *APACHE II* Acute Physiology and Chronic Health Evaluation II, *HMGB1* high mobility group protein 1, *IL-6* interleukin-6, *NSE* neuron specific enolase, *ROSC* return of spontaneous circulation, *sCD24* soluble cluster of differentiation 24, *sSiglec-10* soluble Siglec-10, *TNF-α* tumor necrosis factor-α.
