# Supplementary material for: Circulating CD24/Siglec-10 biomarkers predict post-resuscitation outcomes in a cardiac arrest cohort
Source: Sci Rep. 2025 Oct 29;15:37816. doi: 10.1038/s41598-025-21775-z (PMC12572198; doi:10.1038/s41598-025-21775-z)
Supplement: Supplementary file 2 — Supplementary Material 2 [file 41598_2025_21775_MOESM2_ESM.docx]

|  | **OHCA** | | |  | **IHCA** | | |
| --- | --- | --- | --- | --- | --- | --- | --- |
|  | **Survivors** | **Non-survivors** | ***P*** |  | **Survivors** | **Non-survivors** | ***P*** |
|  | *n*=9 | *n*=24 |  |  | *n*=22 | *n*=49 |  |
| **Age**, years | 66.0 (47.3, 69.8) | 67.5 (48.8, 80.8) | 0.903 |  | 68.0 (65.5, 73.8) | 68.0 (51.8, 76.0) | 0.222 |
| **Male**, *n* (%) | 5 (55.6%) | 15(62.5%) | 0.482 |  | 12 (54.5%) | 32 (65.3%) | 0.321 |
| **Witnessed CA**, *n* (%) | 5 (55.6%) | 0 (0%) | 0.000 |  | 22 (100%) | 49 (100%) | 0.091 |
| **Bystander CPR**, *n* (%) | 6 (66.7%) | 1(4.2%) | 0.000 |  | 22(100%) | 49 (100%) | 0.038 |
| **Initial cardiac rhythm**, *n* (%) |  |  |  |  |  |  |  |
| **Shockable rhythm** | 1 (11.1%) | 2 (8.3%) | 0.808 |  | 18 (81.8%) | 17 (34.7%) | 0.000 |
| **Non-shockable rhythm** | 8 (88.9%) | 22 (91.7%) | 0.808 |  | 4 (18.2%) | 32 (65.3%) | 0.000 |
| **CPR time**, minutes | 5.0 (4.0, 8.8) | 16.5 (12.0**–**25.5) | 0.000 |  | 8.0 (5.0, 11.3) | 15.0 (10.0**–**20.0) | 0.001 |
| **MIRACLE_2_ score** | 3 (2.0, 3.0) | 7 (6.0, 8.0) | 0.000 |  | – | – | – |
| **GO-FAR 2 score** | – | – | – |  | 0 (-3.0, 1.3) | 6 (4.0**–**8.0) | 0.000 |
| **28-day CPC (1–2)**, *n* (%) | 6 (66.7%) | – | – |  | 12 (54.5%) | – | – |

**Supplementary Table S2.** Baseline characteristics of the study participants with OHCA or IHCA. All data are presented as median (interquartile range), unless otherwise specified. *CA* cardiac arrest, *CPC* cerebral performance category, *CPR* cardiopulmonary resuscitation, *GO-FAR 2* the Good Outcome Following Attempted Resuscitation (GO-FAR) 2 score, *IHCA* in- hospital cardiac arrest, *MIRACLE_2_* a risk score for early prediction of neurological outcome after out-of-hospital cardiac arrest, *OHCA* out-of-hospital cardiac arrest.
