# Supplementary material for: Circulating CD24/Siglec-10 biomarkers predict post-resuscitation outcomes in a cardiac arrest cohort
Source: Sci Rep. 2025 Oct 29;15:37816. doi: 10.1038/s41598-025-21775-z (PMC12572198; doi:10.1038/s41598-025-21775-z)
Supplement: Supplementary file 3 — Supplementary Material 3 [file 41598_2025_21775_MOESM3_ESM.docx]

| **Variables** | **AUC** | **95% CI** | ***P*** | **Cut-off**  (ng/mL) | **Specificity**  (%) | **Sensitivity**  (%) | **NPV**  (%) | **PPV**  (%) | **LR–** | **LR+** | **Youden**  (%) |
| --- | --- | --- | --- | --- | --- | --- | --- | --- | --- | --- | --- |
| **sCD24_D1_^OHCA^** | 0.860 | 0.734-0.986 | 0.003 | 7.86 | 100.0 | 80.0 | 50.0 | 100.0 | 0.20 | – | 80.0 |
| **sCD24_D1_^IHCA^** | 0.721 | 0.603-0.839 | 0.003 | 9.33 | 90.9 | 51.0 | 56.5 | 89.3 | 0.54 | 5.61 | 41.9 |
| **IL-6_D1_^OHCA^** | 0.650 | 0.438-0.862 | 0.208 | 69.64 | 50.0 | 84.0 | 50.0 | 84.0 | 0.32 | 1.68 | 34.0 |
| **IL-6_D1_^IHCA^** | 0.767 | 0.648-0.887 | 0.000 | 65.19 | 63.6 | 83.7 | 63.6 | 83.7 | 0.26 | 2.30 | 47.31 |
| **sCD24_D1_ ^OHCA^ + IL-6_D1_^OHCA^** | 0.945^AB^ | 0.871-1.000 | 0.000 | – | 100.0 | 88.0 | 62.5 | 100.0 | 0.12 | – | 88.0 |
| **sCD24_D1_ ^IHCA^ + IL-6_D1_^IHCA^** | 0.858^ab^ | 0.774-0.943 | 0.000 | – | 90.9 | 73.5 | 69.0 | 92.7 | 0.29 | 8.09 | 64.38 |

**Supplementary Table S3.** Areas under the curve and performance of various parameters for predicting the 28-day all‐cause mortality in IHCA and OHCA patients after ROSC. ^A^*P*=0.091 (*Z*=1.688) vs sCD24_D1_; ^B^*P*=0.004 (*Z*=2.864) vs IL-6_D1_; ^a^*P*=0.011 (*Z*=2.555) vs sCD24_D1_; ^b^*P*=0.038 (*Z*=2.071) vs IL-6_D1_; *AUC* area under the curve, *CI* Confidence interval, *IHCA* in-hospital cardiac arrest, *IL-6_D1_^IHCA^* interleukin-6 on day 1 after ROSC in IHCA, *IL-6 _D1_^OHCA^* interleukin-6 on day 1 after ROSC in OHCA, *LR^–^*, negative likelihood ratio, *LR^+^* positive likelihood ratio, *NPV*, negative predictive value, *OHCA* out-of-hospital cardiac arrest, *PPV* positive predictive value, *ROSC* return of spontaneous circulation, *sCD24_D1_^IHCA^* soluble cluster of differentiation 24 on day 1 after ROSC in IHCA, *sCD24_D1_^OHCA^* soluble cluster of differentiation 24 on day 1 after ROSC in OHCA.
