# Supplementary material for: Circulating CD24/Siglec-10 biomarkers predict post-resuscitation outcomes in a cardiac arrest cohort
Source: Sci Rep. 2025 Oct 29;15:37816. doi: 10.1038/s41598-025-21775-z (PMC12572198; doi:10.1038/s41598-025-21775-z)
Supplement: Supplementary file 4 — Supplementary Material 4 [file 41598_2025_21775_MOESM4_ESM.docx]

| **Variables** | **AUC** | **95%CI** | ***P*** | **Cut-off**  (ng/mL) | **Specificity**  (%) | **Sensitivity**  (%) | **NPV**  (%) | **PPV**  (%) | **LR–** | **LR+** | **Youden**  (%) |
| --- | --- | --- | --- | --- | --- | --- | --- | --- | --- | --- | --- |
| **sCD24_D1_^OHCA^** | 0.858^AB^ | 0.729-0.987 | 0.007 | 7.86 | 100 | 74.1 | 46.2 | 100 | 0.26 | – | 74.1 |
| **NSE_D3_ ^OHCA^** | 0.958 | 0.873-1.000 | 0.002 | 26.46 | 100 | 83.3 | 75.0 | 100 | 0.17 | – | 83.3 |
| **MIRACLE_2 D1_^OHCA^** | 0.988 | 0.729-0.987 | 0.000 | 4.50 | 100 | 88.9 | 66.7 | 100 | 0.11 | – | 96.3 |
| **sCD24_D1_^OHCA^ +NSE_D3_^OHCA^** | 1.000^CD^ | 1.000-1.000 | 0.000 | – | 100 | 100 | 100 | 100 | 0.00 | – | 100 |
| **sCD24_D1_^OHCA^ + MIRACLE_2 D1_^OHCA^** | 0.994^EF^ | 0.974-1.000 | 0.000 | – | 96.3 | 100 | 100 | 100 | 0.00 | 27.03 | 96.3 |
| **sCD24_D1_^IHCA^** | 0.806^ab^ | 0.695-0.917 | 0.000 | 2.78 | 75.0 | 78.1 | 41.8 | 93.7 | 0.29 | 3.12 | 53.0 |
| **NSE_D3_^IHCA^** | 0.825 | 0.711-0.939 | 0.001 | 16.24 | 58.3 | 80.0 | 46.7 | 86.5 | 0.34 | 1.92 | 38.3 |
| **GO-FAR 2_D1_^IHCA^** | 0.931 | 0.871-0.991 | 0.000 | 0.50 | 89.8 | 83.3 | 53.0 | 97.5 | 0.19 | 8.17 | 73.2 |
| **sCD24_D1_^IHCA^ +NSE_D3_^IHCA^** | 0.925^cd^ | 0.853-0.997 | 0.000 | – | 83.3 | 90.0 | 66.7 | 95.5 | 0.12 | 5.40 | 73.3 |
| **sCD24_D1_^IHCA^ + GO-FAR 2_D1_^IHCA^** | 0.962^ef^ | 0.919-1.000 | 0.000 | – | 100 | 91.5 | 71.1 | 100 | 0.09 | – | 91.5 |

**Supplementary Table S4.** Areas under the curve and performance of various parameters for predicting the 28-day poor neurological prognosis in OHCA and IHCA patients after ROSC. ^A^*P*=0.485 (*Z*=-0.698) vs. NSE_D3_; ^B^*P*=0.050(*Z*=-1.963) vs. MIRACLE_2 D1_; ^C^*P*=0.190(*Z*=1.311) vs. sCD24_D1_; ^D^*P*=0.313 (*Z*=1.009) vs. NSE_D3_; ^E^*P*=0.035(*Z* =2.103) vs. sCD24_D1_; ^F^*P*=0.398(*Z* =0.845) vs. MIRACLE_2 D1_; ^a^*P*=0.590 (*Z*=0.539) vs. NSE_D3_; ^b^*P*=0.059(*Z*=-1.889) vs. GO FAR 2_D1_; ^c^*P*=0.057 (*Z*=1.904) vs. sCD24_D1_; ^d^*P*=0.032 (*Z*=2.143) vs. NSE_D3_; ^e^*P*=0.004(*Z* =2.862) vs. sCD24_D1_; ^f^*P*=0.116(*Z* =1.574) vs. GO FAR 2_D1_; *AUC* area under the curve, *CI* Confidence interval, *GO-FAR 2* the Good Outcome Following Attempted Resuscitation (GO-FAR) 2 score, *IHCA* in-hospital cardiac arrest, *LR^–^* negative likelihood ratio, *LR^+^* positive likelihood ratio, *MIRACLE_2_* a risk score for early prediction of neurological outcome after out-of-hospital cardiac arrest, *NSE_D3_* neuron specific enolase on day 3 after ROSC, *NPV*, negative predictive value, *OHCA* out-of-hospital cardiac arrest, *PPV* positive predictive value, *ROSC* return of spontaneous circulation, *sCD24_D1_* soluble cluster of differentiation 24 on day 1 after ROSC.
